# When body image and fear collide: a fuzzy-set qualitative comparative (fsQCA) and mediation analysis of sufficient pathways to post-traumatic stress in breast cancer survivors

**DOI:** 10.3389/fpsyg.2026.1769571

**Published:** 2026-04-13

**Authors:** Yifan Gong, Bingnan Du, Minyi Wang, Xinchen Wang, Zhihui Gu, Li Liu, Hui Wu, Mengyao Li

**Affiliations:** Department of Social Medicine, School of Health Management, China Medical University, Shenyang, Liaoning, China

**Keywords:** body image disturbance, breast cancer survivors, Conservation of Resources Theory, fear of progression, fsQCA, posttraumatic stress symptoms

## Abstract

**Background:**

Breast cancer survivors frequently experience significant posttraumatic stress symptoms (PTSS). Using the Conservation of Resources (COR) theory as a framework, this study aimed to investigate the configurational patterns and underlying psychological associations linked to high PTSS in post-operative patients.

**Methods:**

A cross-sectional study was conducted with 172 post-operative breast cancer patients. Participants completed measures for PTSS, resilience, perceived social support (PSS), self-efficacy, fear of progression (FoP), body image disturbance (BID), and shared decision-making (SDM). The analysis employed fuzzy-set Qualitative Comparative Analysis (fsQCA) to identify sufficient combinations of factors associated with high PTSS. This was followed by mediation analysis to test interrelationships among the identified core conditions.

**Results:**

The prevalence of PTSS was 89.5%. fsQCA identified five sufficient configurations for high PTSS. The combination of high BID and high FoP emerged as a core condition across these patterns. Mediation analysis demonstrated that FoP was associated with the link between BID and PTSS, and linked PSS to PTSS.

**Conclusion:**

High FoP and high BID are central factors correlated with PTSS in breast cancer survivors, operating within a COR-based “loss spiral.” Low SDM functions as a conditional factor related to resource obstruction. Findings suggest a need for stratified interventions targeting these specific resource depletions.

## 1 Introduction

Breast cancer (BC) is one of the most prevalent malignancies among women globally, with a rising incidence that poses a significant threat to their health and quality of life (Arnold et al., 2022). Compared to other cancers, its treatment is characterized by a prolonged duration, highly invasive procedures, and frequently involves the surgical removal of gender-defining organs, such as the breast (Waks and Winer, 2019). Furthermore, since the patients are predominantly female, issues such as health perceptions (Oksuzyan et al., 2019) and body image (Öberg and Tornstam, 1999) become particularly salient (Muzzatti et al., 2020). These are critical factors for psychological wellbeing.

This prolonged psychological burden is often associated with posttraumatic stress symptoms (PTSS) (Pitman et al., 2001). PTSS refers to a cluster of psychological reactions occurring after a major traumatic event, including re-experiencing, avoidance, negative alterations in cognitions and mood, and marked alterations in arousal and reactivity (Classen et al., 1998). Research indicates that the prevalence of PTSS among BC patients is 36%, a rate significantly higher than that in the general population (Green et al., 1998). PTSS not only diminishes patients’ quality of life (Harris et al., 2024) but is also linked to adverse health outcomes, such as poor treatment adherence, compromised immune function, an increased risk of disease recurrence, and higher mortality rates (Grassi et al., 2021). Therefore, it is clinically important to investigate the potential predictors correlates and the complex interplay of factors associated with PTSS.

The Conservation of Resources (COR) theory provides a robust framework for understanding PTSS in BC patients. COR theory posits that psychological stress arises from the threatened or actual loss of resources (Hobfoll et al., 1997). In the context of BC, surgical treatments like mastectomy (Platt et al., 2011) and hair loss (Peera et al., 2024) are often associated with a depletion of physical and identity-related resources, and is frequently linked to Body Image Disturbance (BID) (Sun et al., 2018). Conversely, perceived social support (PSS) (Thompson et al., 2017) and shared decision-making (SDM) (Mandelblatt et al., 2006) serve as critical external resources, while self-efficacy (SE) (Zulkosky, 2009) and resilience (RS) act as internal protective resources (Vella and Pai, 2019; Gallagher et al., 2020; Hoge et al., 2007). Furthermore, disease-specific such as fear of progression (FoP) show significantly links to resources depletion (Dinkel and Herschbach, 2018; Guan et al., 2025).

Although previous research has identified predictors of PTSS using variable-centered methods, these approaches often overlook the complex interplay and configurations of multiple conditions. To further elucidate this complexity, the present study employs a hybrid analytical approach. First, we use fuzzy-set Qualitative Comparative Analysis (fsQCA) to identify the configurational patterns (Rihoux and Ragin, 2009; Ragin, 2014). The specific combinations of RS, SE, PSS, BID, SDM, and FoP, that comprise sufficient conditions associated with high levels of PTSS. We examine the concurrent statistical associations among the core conditions identified by fsQCA through an analysis of their interrelationships within a mediation framework. Given the cross-sectional design, this approach characterizes the associations between resources. This provides a nuanced framework for understanding PTSS and informing personalized psychological interventions for BC survivors.

## 2 Materials and methods

### 2.1 Participants

This cross-sectional study was conducted at a cancer hospital in China from April 2022 to September 2023. All data were collected from patients within 1 week following their breast cancer surgery. Inclusion criteria: (1) women aged ≥18 years who were fluent in Chinese; (2) be in the post-surgical recovery phase of BC (3) intact cognition and ability to communicate effectively and complete the questionnaire. Exclusion criteria: (1) pre-existing mental illness, cognitive impairment, or intellectual disability before cancer diagnosis; (2) advanced-stage BC. Written informed consent was obtained from all participants, who volunteered to take part in the study. A total of 210 women were recruited using a consecutive sampling method, and 38 were subsequently excluded, yielding a valid questionnaire rate of 81.9%.

### 2.2 Measures

#### 2.2.1 Socio-demographic and clinical characteristics

Socio-demographic and clinical information was collected through a structured questionnaire and medical records. Socio-demographic data included residence (urban/rural), educational level, marital status, and parity (presence of children). Clinical variables included pathological cancer stage (0–IV), chemotherapy history (yes/no), and surgical method (mastectomy or breast-conserving surgery/reconstruction).

#### 2.2.2 Psychological resilience

RS was assessed with the 14-item Resilience Scale (Wagnild, 2009). Each item is rated on a 7-point Likert scale ranging from 1 (strongly disagree) to 7 (strongly agree); higher total scores indicate greater RS. In the present sample the KMO measure was 0.938 and the single-factor solution explained 60.80% of the total variance. Internal consistency was excellent (Cronbach’s *α* = 0.950).

#### 2.2.3 Perceived social support

PSS was measured with the 12-item Multidimensional Scale of Perceived Social Support (MSPSS) (Zimet et al., 1990). Each item is rated on a 7-point Likert scale from 1 (strongly disagree) to 7 (strongly agree), with higher scores indicating greater perceived support from family, friends, and significant others. In this sample the KMO value was 0.939 and the total variance explained was 71.64%. Internal consistency was excellent (Cronbach’s *α* = 0.964).

#### 2.2.4 Self-efficacy

SE was assessed with the 10-item General Self-Efficacy Scale (GSE) (Zeng et al., 2022). Each item is rated on a 4-point Likert scale from 1 (not at all true) to 4 (exactly true), with higher total scores indicating stronger beliefs in one’s ability to cope with difficult situations. In the present sample the KMO measure was 0.918 and the single-factor solution explained 63.90% of the total variance. Internal consistency was good (Cronbach’s *α* = 0.936).

#### 2.2.5 Fear of progression

FoP was measured with the 12-item Fear of Progression Questionnaire-Short Form (FoP-Q-SF) (Mehnert et al., 2009). Each item is rated on a 5-point Likert scale from 1 (never) to 5 (almost always), with higher scores indicating greater fear of disease progression. In the current sample the KMO value was 0.873 and the total variance explained was 58.83%. Internal consistency was good (Cronbach’s *α* = 0.894).

#### 2.2.6 Body image disturbance

BID was assessed with the 10-item Body Image Scale (BIS) (Hopwood et al., 2001). Each item is rated on a 4-point Likert scale from 0 (not at all) to 3 (very much), with higher total scores indicating greater body image dissatisfaction. In the current sample the KMO value was 0.899 and the total variance explained was 68.15%. Internal consistency was good (Cronbach’s *α* = 0.902).

#### 2.2.7 Shared decision-making

SDM was assessed with the 9-item Shared Decision-Making Questionnaire (SDM-Q-9) (Calderon et al., 2018). Each item is rated on a 6-point Likert scale from 0 (strongly disagree) to 5 (strongly agree), with higher scores indicating greater perceived shared decision-making. In the current sample the KMO value was 0.913 and the total variance explained was 83.57%. Internal consistency was good (Cronbach’s *α* = 0.974).

#### 2.2.8 Posttraumatic stress symptoms

PTSS were measured with the 22-item Impact of Event Scale-Revised (IES-R) (Hyer and Brown, 2008). Participants rated how much each symptom had distressed them in the past week on a 5-point scale from 0 (not at all) to 4 (extremely). A total score was calculated by summing the scores of all items, resulting in a possible range of 0–88. Based on the total score, symptom severity was categorized as follows: 0–8 indicated no clinically significant symptoms, 9–25 indicated mild symptoms, 26–43 indicated moderate symptoms, and a score of 44 or higher indicated severe symptoms. Higher total scores are indicative of greater severity of PTSS. In this sample the KMO value was 0.946 and the total variance explained was 69.75%. Internal consistency was excellent (Cronbach’s *α* = 0.965).

### 2.3 Statistical procedures

Data were analyzed using SPSS Statistics (v.25.0) and the fsQCA software (v.3.0). Prior to the main analyses, descriptive statistics, including frequencies and percentages, were calculated to summarize the sociodemographic and clinical characteristics of the participants.

Our primary analysis followed a two-stage approach, consistent with the study’s objective to move from a configurational to a comprehensive understanding of the interrelationships associated with PTSS. The first stage employed fsQCA to identify the complex combinations of antecedent conditions that consistently related to high/low levels of PTSS. This procedure involved three main steps: (1) calibrating the antecedent conditions and the outcome variable into fuzzy-set scores ranging from 0 (full non-membership) to 1 (full membership); (2) conducting an analysis of necessity to determine if any single condition was essential for the presence of the outcome; and (3) performing a sufficiency analysis via a truth table to identify the distinct configurational patterns associated with high PTSS. Robustness checks were also conducted to ensure the stability of the final configurational solution.

In the second stage, we focused on exploring the potential mediating associations connecting the key factors identified in the fsQCA. Based on the core conditions that emerged as central components of the high-PTSS configurations, a mediation analysis was performed. This targeted approach allowed for a more theoretically grounded and parsimonious model. Specifically, we used the PROCESS macro (v.3.2, Model 4) for SPSS, developed by Hayes, to examine the mediating roles among these empirically identified core conditions and their statistical associations with PTSS.

Given that all data were collected from a single institution, Harman’s single-factor test was performed to assess potential common method bias. An exploratory factor analysis involving all key study variables was conducted for this purpose. The results showed that the first unrotated factor accounted for 24.91% of the total variance, which is below the recommended threshold of 50%. This suggests that common method bias was not a significant concern in this study (Harrison et al., 1996).

## 3 Results

### 3.1 Demographic characteristics

Among the 172 participants, 71.5% resided in urban areas and 88.4% were married or cohabiting. In terms of clinical profiles, most patients were diagnosed at Stage II (81.4%) and had received chemotherapy (90.7%). Notably, 73.3% of the sample underwent a mastectomy, while 26.7% received breast-conserving surgery or reconstruction. Detailed demographic and clinical characteristics are presented in Table 1.

**Table 1:** Basic demographic characteristics of the participants (*n* = 172).

Items	Type	*n*	Percentage
Residence	Urban	123	71.5%
	Rural	49	28.5%
Educational attainment	Junior high school and below	85	49.4%
	High school/secondary specialized school	49	28.5%
	College/University	36	20.9%
	Postgraduate and above	2	1.2%
Marital status	Single/divorced	20	11.6%
	Married/cohabiting	152	88.4%
Children	Yes	166	96.5%
	No	6	3.5%
Cancer stage	0–I	6	3.5%
	II	140	81.4%
	III/IV	26	15.1%
Chemotherapy	Yes	156	90.7%
	No	16	9.3%
Surgery method	Mastectomy	126	73.3%
	Breast-conserving surgery/reconstruction	46	26.7%
PTSS	No clinically significant symptoms	18	10.5%
	Mild symptoms	77	44.8%
	Moderate symptoms	38	22.1%
	Severe symptoms	39	22.7%

### 3.2 Descriptive statistics

#### 3.2.1 Descriptive statistics and intercorrelations

RS, PSS, SE and SDM all correlated positively with one another, whereas FoP, BID and PTSS moved in the opposite direction (see Table 2).

**Table 2:** Descriptive statistics and intercorrelations.

Variables	1	2	3	4	5	6	7
1. RS	1						
2. PSS	0.517**	1					
3. SE	0.500**	0.316**	1				
4. FoP	−0.318**	−0.260**	−0.320**	1			
5. BID	−0.230**	−0.259**	−0.280**	0.497**	1		
6. SDM	0.192*	0.223**	0.174*	−0.204**	−0.253**	1	
7. PTSS	−0.158*	−0.221**	−0.165*	0.499**	0.542**	−0.226**	1

**p < 0.01, *p < 0.05.

#### 3.2.2 Multiple regression models

Only FoP (*B* = 0.623, *p* < 0.001) and BID (*B* = 1.170, *p* < 0.001) emerged as unique predictors, each accounting for roughly a third of a standard-deviation increase in the outcome. No other variable reached significance (all *p* > 0.05). More details are provided in Table 3.

**Table 3:** The results of the multiple linear regressions.

Variables	*B*	S.E	*β*	*t*	*p*
Intercept	−4.551	10.180		−0.447	0.655
RS	0.071	0.106	0.053	0.667	0.505
PSS	−0.089	0.094	−0.069	−0.938	0.349
SE	0.121	0.188	0.047	0.645	0.520
FoP	0.623	0.148	0.312	4.223	**<0.001**
BID	1.170	0.227	0.377	5.153	**<0.001**
SDM	−0.110	0.103	−0.070	−1.073	0.285

*B*, estimated coefficient; S.E., standard error; *β*, standardized regression coefficients.

Bold values indicate that the variable is significant in the regression.

### 3.3 Fuzzy set qualitative comparative analysis model (fsQCA)

#### 3.3.1 Calibration

As a prerequisite for the analysis of necessary and sufficient conditions, a calibration of the raw variable data was conducted. This procedure involved converting the original measurements into fuzzy membership scores on a continuous scale from 0 (fully out of the set) to 1 (fully in the set). This transformation was anchored by three critical thresholds derived from the data’s distribution: the 75th percentile defined full membership, the 50th percentile marked the point of maximum ambiguity (the crossover point), and the 25th percentile defined full non-membership. A summary of the variables and their calibration anchors is presented in Table 4.

**Table 4:** Statistical descriptions and calibration values.

Summary statistics	RS	PSS	SE	FoP	BID	SDM	PTSS
*M*	73.03	63.66	25.15	34.43	10.53	34.45	27.99
*SD*	12.09	12.49	6.24	8.01	5.16	10.11	16.02
Min	30	26	11	12	0	0	0
Max	98	84	40	60	27	45	85
Calibration values							
P_25_	64.25	52.00	20.00	30.00	8.00	27.00	18.00
P_50_	74.00	65.50	25.00	36.00	10.00	36.00	23.50
P_75_	83.00	72.00	30.00	38.75	13.00	45.00	40.00

*M*, Means; SD, Standard Deviations; Min, minimum; Max, maximum; P_25_, 25th percentile; P_50_, 50th percentile; P_75_, 75th percentile.

#### 3.3.2 Necessity analysis

The analysis revealed no necessary conditions for the outcome. Based on the 0.90 consistency benchmark (Schneider et al., 2010), Table 5 shows that neither high nor low PTSS was a necessary condition.

**Table 5:** Results of the necessity analysis of high and low PTSS.

Condition variable	Outcome			
	High-PTSS		Low-PTSS	
	Consistency	Coverage	Consistency	Coverage
RS	0.482066	0.492029	0.582214	0.589156
~RS	0.597476	0.590576	0.498015	0.488046
PSS	0.523793	0.494481	0.611468	0.572303
~PSS	0.546949	0.586759	0.459886	0.489132
SE	0.515677	0.541277	0.542754	0.564817
~SE	0.585400	0.563574	0.559196	0.533734
FoP	0.673961	0.727380	0.406248	0.434691
~FoP	0.476207	0.447196	0.745218	0.693823
BID	0.734086	0.699975	0.463576	0.438247
~BID	0.410872	0.435846	0.682635	0.717923
SDM	0.475651	0.482285	0.643116	0.646498
~SDM	0.651361	0.647999	0.484994	0.478357

~ refers to negation. In this study, ~ X was considered to be a lower level of X.

#### 3.3.3 Sufficiency analysis

The sufficiency analysis in this research employed a truth table constructed with Boolean logic, providing a clear framework to assess every possible logical combination and its corresponding result. In line with Ragin (2006) recommendations for balancing precision with parsimony in causal reasoning, we established a consistency threshold of 0.8. This benchmark signifies that a causal path is highly stable and dependable in its connection to the outcome. To further bolster the reliability of our findings and avoid conclusions drawn from an insufficient number of examples, a case threshold of 2 was set, a suitable figure for our sample size. A minimum PRI consistency of 0.70 was also required. The computational process produced three solutions: parsimonious, intermediate, and complex. We then distinguished between core and peripheral conditions based on these results. Core conditions were defined as those appearing in both the parsimonious and intermediate solutions, whereas peripheral conditions were those found exclusively in the intermediate solution.

#### 3.3.4 Configurations for achieving the high PTSS

As detailed in Table 6, our analysis identified five configurations leading to high PTSS in BC patients. Solution consistency is required to exceed 0.75, with coverage above 0.25 (Brown, 2009). This demonstrates strong explanatory power. Specifically, these five pathways account for 45.7% of all instances of high PTSS in the sample. Moreover, they are highly reliable predictors, as 83.8% of the cases fitting these configurations reported high PTSS outcomes.

**Table 6:** Configurations for achieving the high posttraumatic stress symptoms (fsQCA).

Condition variable	High-PTSS				
	H1	H2	H3	H4	H5
RS	ⓧ			●	●
PSSS	●			●	ⓧ
SE			●	●	ⓧ
FoP	●	●	●	●	●
BIS	●	●	●	●	●
SDM	ⓧ	ⓧ	ⓧ		●
consistency	0.846674	0.846733	0.882936	0.845266	0.845266
raw coverage	0.303115	0.213639	0.262765	0.149265	0.0604493
unique coverage	0.0778744	0.0236309	0.0238625	0.0334839	0.0128633
solution consistency	0.83825				
solution coverage	0.457277				

Large ● indicates the inclusion of a factor as a core factor in this structure; Small • indicates the inclusion of a factor as an auxiliary factor in this structure (edge conditions exist); Large ⓧ indicates that the core factor is not included in this structure (the core condition is absent); Small ⓧ indicates that auxiliary factors are not included in this structure (the marginal condition is absent); The blank indicates that the variable may or may not be present in the configuration.

#### 3.3.5 Configurations for achieving the low PTSS

Turning to the outcome of low PTSS, Table 7 outlines four distinct causal pathways. The model’s overall consistency is 0.841, meaning 84.1% of cases encompassed by these configurations had low PTSS. The overall coverage is 0.415, indicating that these paths account for 41.5% of all observed instances of low PTSS.

**Table 7:** Configurations for achieving the low posttraumatic stress symptoms (fsQCA).

Condition variable	Low-PTSS			
	L1	L2	L3	L4
RS	ⓧ		●	●
PSSS	ⓧ	ⓧ	•	ⓧ
SE	ⓧ	•		ⓧ
FoP		ⓧ	ⓧ	ⓧ
BIS	ⓧ	ⓧ	ⓧ	ⓧ
SDM	●	●	●	ⓧ
consistency	0.857815	0.835002	0.891412	0.846154
raw coverage	0.149083	0.124699	0.262198	0.0655145
unique coverage	0.0687491	0.0332711	0.18991	0.0158823
solution consistency	0.840573			
solution coverage	0.415123			

Large ● indicates the inclusion of a factor as a core factor in this structure; Small • indicates the inclusion of a factor as an auxiliary factor in this structure (edge conditions exist); Large ⓧ indicates that the core factor is not included in this structure (the core condition is absent); Small ⓧ indicates that auxiliary factors are not included in this structure (the marginal condition is absent); The blank indicates that the variable may or may not be present in the configuration.

#### 3.3.6 Robustness analyses

We tested the robustness by increasing the case frequency threshold from 2 to 3. The resulting configurations were subsets of the initial solutions. According to Schneider and Wagemann (2012), this subset relationship indicates that the core causal structures are stable and the findings are robust (Supplementary Table 2). Raising the consistency threshold from 0.80 to 0.85 left the core configurations unchanged (solution consistency > 0.81; coverage > 0.37), confirming robustness (White et al., 2021).

### 3.4 Mediation effect analysis

**Figure 1:**
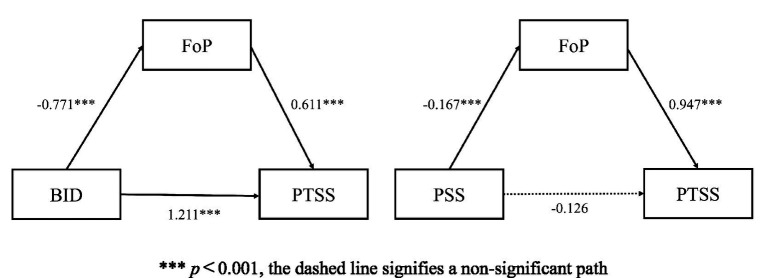
Mediation analysis results. ***p < 0.001, the dashed line signifies a non-significant path.

The mediation analysis reveals two distinct pathways. In model 2, FoP serves as a full mediator between PSS and PTSS, as indicated by a significant indirect effect (ab = −0.158) and a non-significant direct effect (c’ = −0.126). Conversely, in model 1, FoP acts only as a partial mediator between BID and PTSS. For this pathway, both the indirect effect (ab = 0.471) and the direct effect (c’ = 1.211) are significant (see Table 8 and Figure 1).

**Table 8:** Results of the mediation effect analysis.

Model	a	b	c’	c	ab [95%CI]
1. BID → FOP → PTSS	0.771***	0.611***	1.211***	1.682***	0.471 [0.207, 0.793]
2. PSS → FOP → PTSS	−0.167***	0.947***	−0.126	−0.283**	−0.158 [−0.271, −0.060]

***p < 0.001, **p < 0.01, CI, Confidence Interval.

## 4 Discussion

### 4.1 Principal findings

A key finding is the high prevalence of PTSS (89.5%) among post-operative BC patients. This rate appears to exceed the prevalence reported in some previous meta-analyses and studies, suggesting that the psychological impact of BC may be even more pervasive than previously understood (Swartzman et al., 2017; Pranjic et al., 2016). This high prevalence may be associated with our sample’s characteristics, including the type of surgery and the timing of assessment (Andrykowski and Cordova, 1998). 73.3% of the participants underwent a mastectomy, and all surveys were conducted within 1 week post-surgery. During this specific period, patients typically experience heightened psychological stress and maintain a high level of concern regarding their physical status. This significant finding highlights an urgent need for proactive mental health screening, as a large number of patients are clearly suffering from significant distress that requires clinical attention.

Our fsQCA results identified the combination of high BID and high FoP as a core sufficient condition associated with high PTSS. Conversely, the joint absence of these factors was a core element in most pathways for low PTSS, highlighting their synergistic relationship.

Although medical advancements have lowered recurrence rates (van Maaren et al., 2019), patients receive this information in “probabilistic language” but experience it as a “catastrophic narrative”—any disease-related information can trigger automatic thoughts like “I’ll be next” (Chae, 2016). Our finding that FoP is a core condition aligns with biological evidence. For instance patients with high FoP show high HPA axis blunting similar to these with PTSD (Algamal et al., 2021). This suggests that FoP may not be merely an emotional state but may also be closely linked to a trauma-like physiological pathway. In Chinese cultural context, societal expectation of motherhood may intensify the fear of recurrence with creating family risk (Fan et al., 2014). This aligns with our observation that FoP remains a stable factor for PTSS (Jin et al., 2021).

In this study, 73.3% of participants underwent a mastectomy, and all assessments were conducted within the first post-operative week. At this specific juncture, patients typically experience heightened psychological stress and maintain a high level of concern regarding their physical status. The high level of BID observed in our study may reflect a disruption of the patient’s self-identity. Mastectomy involves more than the objective loss of tissue. This process can be viewed as a form of “symbolic death” (it denotes the extinction of the individual’s “authentic self” or subjectivity, rather than physical death) (Piot-Ziegler et al., 2010). Qualitative study such as “the breast is the organ that shows women as beautiful” and “When I look in the mirror, my breast is not there. I don’t want to look in the mirror” are highly consistent with the “permanently damaged self” schema in PTSD (Koçan and Gürsoy, 2016; Arnaboldi et al., 2014). 90.7% of the participants completed neoadjuvant chemotherapy prior to surgery. Many participants had already experienced stressors, such as hair loss, before surgery. Scars and hair loss act as persistent visual and tactile triggers. These symptoms are often “re-experienced” during daily activities like dressing or bathing (Voigt et al., 2017). It is worth noting that modern oncological advancements, such as breast-conserving surgery and reconstruction techniques, are increasingly mitigating these physical and psychological burdens (Zhang et al., 2025; Satake et al., 2018). However, for patients who undergo extensive surgery, the alteration in physical appearance remains a significant source of psychological distress during the early recovery phase.

Clinical observation reveals that many patients, due to limited medical knowledge, delegate treatment decisions to physicians, resulting in an asymmetric dynamic where doctors unilaterally decide on surgical methods or chemotherapy regimens (Bickell et al., 2009). Such passivity could be associated with increased uncertainty regarding treatment efficacy and side effects, which in turn might amplify FoP. Concurrently, patients often passively accept mastectomy or hair-loss-inducing chemotherapy without adequate psychological preparation, potentially contributing to a sudden and acute identity crisis post-surgery concerning their “loss of female symbolism,” which may further elevate BID (Halkett et al., 2018). From the COR perspective, FoP (a threatened resource loss), BID (an actual and symbolic resource loss), and low SDM (a barrier to conditional resources) collectively represent a configuration of resource loss. This may continuously deplete emotional, cognitive, and social support reserves, making it difficult for individuals to gain resources through reinvestment and potentially trapping individuals in a self-amplifying loss spiral.

### 4.2 Theoretical implications

This study extends the COR framework by identifying the specific resources depleted in BC patients. We move beyond generic concepts of “personal” or “energy” resources to identify symbolic resources and conditional resources as the primary domains of resource loss within the cancer experience. Furthermore, while COR theory describes “loss spirals” resulting from concurrent threats, it does not explain how these losses combine. Our findings reveal a configurational, rather than additive, logic: the resource threat/loss (BID), and resource gap escalation (FoP) forms a sufficient condition for trauma.

In essence, this research suggests that BID and FoP are not merely side effects, but are central factors associated with the resource depletion described in COR theory. This reframing provides a precise and actionable target for the advancement of precision psychosocial oncology.

### 4.3 Practical implications

Clinical interventions should be structured as a “stepped and integrated” plan: (1) Pre-surgery, introduce visual risk communication and decision aids to enhance SDM and supplement conditional resources (Riganti et al., 2024; Hassan et al., 2024). (2) Within 2 weeks post-surgery, implement mirror exposure (Hao et al., 2025) combined with online mindful self-compassion training (Chen et al., 2024) to repair body image disturbance. (3) One week before follow-up appointments, apply uncertainty tolerance (Blom et al., 2023) and cognitive reappraisal techniques (Li et al., 2015) to weaken the catastrophic processing of FoP and interrupt the loss spiral.

### 4.4 Limitations

This study has several limitations. First, given the cross-sectional nature of the data, the mediation results should be interpreted as exploratory statistical pathways rather than causal mechanisms. Second, the sample was recruited from a single hospital, so caution is needed when generalizing the findings to BC survivors in community or rural settings. Third, fsQCA is sensitive to sample size; future research should use larger samples and longitudinal data to enhance the stability of the configurations.

## 5 Conclusion

High FoP and high BID are central factors associated with PTSS in patients with BC. Low SDM appears to function as a conditional factor linked to resource obstruction, contributing to a pattern of “multiple resource depletion” when combined with the other two factors. The Conservation of Resources theory provides an integrative framework for understanding this loss spiral and may help identify specific targets for the development of precise, stratified psychological interventions.

## Data Availability

The raw data supporting the conclusions of this article will be made available by the authors without undue reservation.
