# Supplementary material for: When body image and fear collide: a fuzzy-set qualitative comparative (fsQCA) and mediation analysis of sufficient pathways to post-traumatic stress in breast cancer survivors
Source: Front Psychol. 2026 Apr 13;17:1769571. doi: 10.3389/fpsyg.2026.1769571 (PMC13111568; doi:10.3389/fpsyg.2026.1769571)
Supplement: Supplementary file 1 [file Supplementary_file_1.docx]

Supplementary Table 1. Means and Standard Deviations of Study Variables

| Variables | *n* | Min | Max | *M* | *SD* |
| --- | --- | --- | --- | --- | --- |
| 1.RS | 172 | 30.00 | 98.00 | 73.03 | 12.09 |
| 2.PSS | 172 | 26.00 | 84.00 | 63.66 | 12.49 |
| 3.SE | 172 | 11.00 | 40.00 | 25.15 | 6.24 |
| 4.FoP | 172 | 12.00 | 60.00 | 34.43 | 8.01 |
| 5.BID | 172 | .00 | 27.00 | 10.53 | 5.16 |
| 6.SDM | 172 | .00 | 85.00 | 27.99 | 16.02 |
| 7.PTSS | 172 | 30.00 | 98.00 | 73.03 | 12.09 |

|  | Variables | consistency | raw coverage | unique coverage | solution consistency | solution coverage |
| --- | --- | --- | --- | --- | --- | --- |
| High - PTSS | 1. ~ RS*~ PSSS* FoP* BID*~ SDM | 0.847990 | 0.241044 | 0.161966 | 0.834472 | 0.414646 |
|  | 2. PSSS*~SE* FoP* BID*~ SDM | 0.853387 | 0.140917 | 0.0546603 |  |  |
|  | 3. RS* PSSS* SE* FoP* BID | 0.845422 | 0.150075 | 0.0844277 |  |  |
|  | 4. RS*~ PSSS*~ SE* FoP* BID* SDM | 0.841996 | 0.061144 | 0.0140211 |  |  |
| Low - PTSS | 1. ~ PSSS* SE*~ FoP*~ BID* SDM | 0.835002 | 0.124699 | 0.0650237 | 0.868035 | 0.328389 |
|  | 2. RS* PSSS*~ FoP*~ BID* SDM | 0.891842 | 0.263366 | 0.203690 |  |  |

Supplementary Table 2. Robustness analyses

~ refers to negation. In this study, ~ X was considered to be a lower level of X.
